# Medical insurance payment schemes and patient medical expenses: a cross-sectional study of lung cancer patients in urban China

**DOI:** 10.1186/s12913-023-09078-3

**Published:** 2023-01-26

**Authors:** Hanxu Hu, Liying Zhao, Yang Yong, Stephen Nicholas, Elizabeth Maitland, Weihan Zhao, Hao Yan, Yong Ma, Xuefeng Shi

**Affiliations:** 1 School of Management, Beijing University of Chinese Medicine, Beijing, China; 2 Medical Device Regulatory Research and Evaluation Center, West China Hospital, Sichuan University, Chengdu, China; 3 Australian National Institute of Management and Commerce, Sydney, NSW Australia; 4 Guangdong Institute for International Strategies, Guangdong University of Foreign Studies, Guangzhou, China; 5 School of Economics and School of Management, Tianjin Normal University, Tianjin, China; 6 Newcastle Business School, University of Newcastle, Callaghan, NSW Australia; 7 University of Liverpool Management School, University of Liverpool, Liverpool, UK; 8 China Health Insurance Research Association, Beijing, China; 9 National Institute of Traditional Chinese Medicine Strategy and Development, Beijing University of Chinese Medicine, Beijing, China

**Keywords:** Payment method, Hospital expenses, Out-of-pocket expenses, Lung cancer

## Abstract

**Background:**

As the main cause of cancer death, lung cancer imposes seriously health and economic burdens on individuals, families, and the health system. In China, there is no national study analyzing the hospitalization expenditures of different payment methods by lung cancer inpatients. Based on the 2010–2016 database of insured urban resident lung cancer inpatients from the China Medical Insurance Research Association (CHIRA), this paper aims to investigate the characteristics and cost of hospitalized lung cancer patient, to examine the differences in hospital expenses and patient out-of-pocket (OOP) expenses under four medical insurance payment methods: fee-for-service (FFS), per-diem payments, capitation payments (CAP) and case-based payments, and to explore the medical insurance payment method that can be conducive to controlling the cost of lung cancer.

**Method:**

This is a 2010–2016, 7-year cross-sectional study. CHIRA data are not available to researchers after 2016. The Medical Insurance Database of CHIRA was screened using the international disease classification system to yield 28,200 inpatients diagnosed with lung cancer (ICD-10: C34, C34.0, C34.1, C34.2, C34.3, C34.8, C34.9). The study includes descriptive analysis and regression analysis based on generalized linear models (GLM).

**Results:**

The average patient age was 63.4 years and the average length of hospital stay (ALOS) was 14.2 day; 60.7% of patients were from tertiary hospitals; and 45% were insured by FFS. The per-diem payment had the lowest hospital expenses (RMB7496.00/US$1176.87), while CAP had the lowest OOP expenses (RMB1328.18/US$208.52). Compared with FFS hospital expenses, per-diem was 21.3% lower (95% CI = -0.265, -0.215) and case-based payment was 8.4% lower (95% CI = -0.151, -0.024). Compared with the FFS, OOP expenses, per-diem payments were 9.2% lower (95% CI = -0.130, -0.063) and CAP was 15.1% lower (95% CI = -0.151, -0.024).

**Conclusion:**

For lung cancer patients, per-diem payment generated the lowest hospital expenses, while CAP meant patients bore the lowest OOP costs. Policy makers are suggested to give priority to case-based payments to achieve a tripartite balance among medical insurers, hospitals, and insured members. We also recommend future studies comparing the disparities of various diseases for the cause of different medical insurance schemes.

## Background

Globally, lung cancer is the second most common cancer, the main cause of cancer in men, and a leading cause of cancer death [1]. Over the last decade, smoking, involuntary smoking and occupational exposure have led to a significant increase in morbidity of lung cancer in China, accounting for more than one-third of all new global lung cancer cases, and China is on track to have the highest number of lung cancer patients in the world by 2025 [2, 3]. The increase in the number of new cancer cases not only challenges China’s public health system, but also imposes a heavy economic burden on families [4]. Between 2013 and 2016, direct medical costs for urban lung cancer in China were estimated at RMB190.8 billion (US$3.03 billion) per year [4], with average medical expenses for lung cancer patients US$13,173 and out-of-pocket (OOP) expenses of US$6768 [5]. These were significant costs when China's per capita GDP was US$11,300 in 2020 [6], which explains how catastrophic medical expenses plunge families into poverty. How China’s medical insurance schemes compensate lung cancer patients is not only important for understanding the medical costs facing patients suffering from China’s second most common cancer, but has implications for medical insurance compensation for patients suffering from other cancers.

Other countries faced similar lung cancer costs, with the per capita annual medical expenditure of U.S. cancer patients four times that of non-cancer patients [7]. The five-year cost of U.S. lung cancer patient care accounts for about 20% of the total cost of cancer care for private health insurers, estimated at US$4.2 billion [8]. China has taken a series of measures, including fundamental reforms to the health system and the establishment of compulsory universal basic medical insurance, to reduce the medical burden on families and hospitals [9, 10]. In 2021, 96% of the population was covered either the government promoted Urban Employee Basic Medical Insurance (UEBMI) or the Urban and Rural Residents Basic Medical Insurance (URRBMI) [11]. The National Medical Insurance Bureau (NMIB) regulated the UEBMI and URRBMI. UEBMI was launched in 1998 to provide benefits for urban employees and urban retired. URRMBI is the amalgamation of the Urban Resident Basic Medical Insurance (URBMI), a subsidized contributory scheme launched in 2007 to provide medical insurance for urban non-employed residents, students, and children and the New Rural Cooperative Medical System (NCMS) formed in 2003 for rural workers and nonworkers. All the insurance schemes are based on government subsidies and member contributions. Guidelines on the integration of URBMI and NCMS were issued in 2016, with URRBMI bringing URBMI benefits and coverage to rural NCMS members [12]. Given the absence of data on URRBMI, we studied urban members whose health insurance system was UEBMI or URBMI.

Basic medical insurance was mainly coordinated at the prefecture-level, and the payment methods varied by province. While basic medical insurance meant lung cancer out-of-pocket (OOP) medical expenses were reduced, the different fund arrangements saw OOP expenses vary by the medical insurance scheme and geographical region [13–15]. Within the basic medical insurance schemes, different insurance payment methods affect the supply and demand for medical services and the funding of health resources. From 1999 to 2008, the main payment methods were the fee-for-service (FFS) and per-diem payment. In 2009, capitation payments (CAP) and case-based payment were proposed and in 2016, diagnostic-related group (DRG) payments were added [16]. There are two main ways of classifying these insurance payment methods, post-payment system and pre-payment system. The pre-payment system means that before the hospital provides medical services, the NMIB pays them in advance according to a negotiated contract, where the payment amount is not directly related to the actual medical cost [17]. The post-payment system refers to the payment of medical expenses to hospitals or patients according to medical expenses after the hospital provides medical services [18]. Medical insurance payments also determine whether, and how many, medical services are provided [19], such as length of stay (LOS) [20], and the number of services [20]. Table 1 sets out China’s insurance payment system, including (FFS), per-diem payment, CAP, and case-based payment [19]. As China's DRG payment in 2011–2016 is still in the exploration stage, DRG data are not available in our dataset.

**Table 1:** Four basic payment methods of medical insurance

Payment Method	Unit of Payment	Form of payment	Comment
1.Fee-for-service	Per service	Post-payment	Separate payments are often made for multiple services per day
2.Per-diem payment	Per visit/bed day/day	Pre-payment	Pay the cost according to the preset service unit
3.Capitation payment	Per beneficiary	Pre-payment	The number of insured and the reimbursement standard are fixed
4.Case-based payment	Per episode	Pre-payment	Packing payment during hospitalization

The payment methods planned by NMIB varies according to different regions, policies, and medical insurance funds. The detail health insurance payment approach is mainly decided by the local NMIB after consulting with related hospitals [16, 21]. FFS is the most common payment method. NMIB compensates medical institutions according to the price and quantity of medical services set in advance, such as inspection, drugs, and surgery [22]. This has made it easier to manage fees for service and facilitates the development of new technologies, but may lead to more expenses for patients and more profits for hospitals. FFS could lead hospitals to over-service patients, increasing patient medical expenses and wasting public health resources [23, 24].

Per-diem payment divides medical services into a unit (including per visit, bed days, and LOS), where the expenses standard for a unit is established through historical data, and NMIB reimburses hospitals according to unit volume. Since per-diem payment is a fixed payment standard, it can cover the expenses of hospital operations, and cover almost all diseases, so that hospitals find it easy to operate and patients easy to understand the payment system. But, as a limited payment method for medical services, it limits the development of new technologies [25, 26]. CAP is paid to the hospital by the health insurance fund based on a fixed per capita quota. When the actual cost exceeds the budget, the hospital bears the additional cost; when costs are below budget, the hospital retains the surplus as profit [24]. CAP can promote cost control and improve the efficiency of the medical insurance system, but it can lead to under-servicing, lower-quality hospital services and negatively impact medical services [27, 28]. The case-based payment compensates the hospital through the predetermined disease payment standard. NMIB have formulated lung cancer case-based payment standards, such as patient access conditions and reimbursement standards. More than one-fifth of Chinese hospitals had implemented case-based payment for inpatient services by 2007, covering common diseases, such as acute appendicitis or hysteromyoma [29, 30].

With its own risks, coverage, financing policies, treatments, medical insurance catalogues, the management of designated hospitals and fund management [19], each payment method results in different hospital and OOP expenses, but all medical insurance payment methods have reduced average hospital expenses [15, 31]. Previous studies on the economic burden of lung cancer have focused on regional costs, medical insurance types and influencing factors, such as smoking and air quality [32–34], but this is the first study of the relationship between lung cancer expenses and alternative insurance payment methods. In this paper, we aim to analyze the characteristics of hospitalized lung cancer patient, to examine disparities of hospital expenses of four payment methods on lung cancer, we try to explore a medical insurance payment method that could be conducive to controlling the cost of lung cancer more efficiently.

## Method

### Data source

Inpatient data were provided by the China Medical Insurance Research Association (CHIRA), which is only available up to 2017 for researchers. Given the local and regional nature of China’s social health insurance system, pre-2017 coverage and benefits have remained broadly constant for URBMI and UEBMI, with our results providing good insight into the efficacy of current social medicine payment systems. The CHIRA provided a randomly stratified 5% UEBMI and URBMI medical insurance database. From January 2010 to December 2016, the data included demographic information, hospital level (primary, secondary and tertiary), hospital expenses and payment methods. Medical expenses were based on medical reimbursement insurance records, focusing on per-visit hospital expenses. The cost indicators of this study included hospital expenses, which were composed of reimbursement expense and OOP expenses within the scope of each medical insurance scheme, and self-pay outside the medical insurance. The types of hospital expenses were divided into diagnosis and treat expenses, conventional (non-TCM) medication expenses and traditional Chinese medicine (TCM) expenses. Our sample consisted of 28,200 patients with lung cancer, who came from 23 provinces and 3 municipalities (Beijing, Tianjin, and Chongqing). According to the International Classification of Diseases (ICD-10), the patient's lung disease diagnosis code was C34, C34.0, C34.1, C34.2, C34.3, C34.8, and C34.9. The diagnosis of lung cancer was performed by clinicians according to the Chinese Guidelines on the Diagnosis and Treatment of Primary Lung Cancer (2011 version to 2016 version). The number of comorbidities combined with secondary and other diagnosis was used as an indicator reflecting the severity a patient 's illness according to previous research [35]. We divided the number of comorbidities into 0,1 and ≥ 2 based on secondary and other diagnosis diagnoses.

### Measures and variables

The dependent variable was the natural logarithm of hospital expenses and OOP expenses per visit. The independent variables were the four payment methods. Control variables comprised sex, age, LOS, type of medical insurance (UEBMI or URBMI), hospital level (primary, secondary and tertiary), number of comorbidities (0–2 +), geographical region (eastern, central, and western) and year (2010–2016). The average RMB to US$ exchange rate was 0.157 from 2010 to 2016.

### Statistical analysis

Descriptive statistics (percentage, mean and standard deviation), median and quartile spacing and Kruskal–Wallis test were used to assess the demographic information and expenses. A generalized linear model (GLM), combined with gamma distribution and logarithmic link function, was used to evaluate the relationship between payment methods, medical expenses and OOP expenses [36]. All statistical analyses were performed using Stata16.0 (StataCorp College Station, TX 77,845 USA), and statistical significance was defined as *P* < 0.05.

## Results

Table 2 shows the basic characteristics of hospitalized lung cancer patients. Among the 28,200 patients, 18,429 (65.4%) were male. The average length of stay (ALOS) was 14.2 days; 74.5% of the patients participated in UEBMI; and 60.7% were inpatients at tertiary hospitals. Among the four payment methods, FFS (45.0%) and per-diem payment (41.5%) were the dominant schemes, followed by CAP (11.6%) and case-based payments (1.9%). Most inpatients (89.8%) had no comorbidity, and only 6.1% of the patients had two or more diseases. About sixty percent (58.6%) of inpatients were from the central region.

**Table 2:** Sample characteristics of inpatients with lung cancer

Characteristics	N (%)
Sex	
Male	18 429(65.4)
Female	9771(34.6)
Age (Mean ± SD)	
63.37 ± 10.62	
Age group	
≤ 39	447(1.6)
40–49	2442(8.7)
50–59	6647(23.6)
60–69	10 338(36.6)
≥ 70	8326(29.5)
Length of stay	
14.23 ± 13.33	
Insurance type	
UEBMI	21 002(74.5)
URBME	7198(25.5)
Hospital level	
Primary	1310(4.6)
Secondary	9777(34.7)
Tertiary	17 113(60.7)
Payment method	
Fee-for-service	12 690(45.0)
Per-diem payment	11 708(41.5)
Capitation payment	3255(11.6)
Case-based payment	547(1.9)
Number of comorbidities	
0	25 310(89.7)
1	1180(4.2)
≥ 2	1710(6.1)
Region	
East	4312(15.3)
Central	16 521(58.6)
West	7367(26.1)
Year	
2010	203(0.7)
2011	84(0.3)
2012	374(1.3)
2013	4602(16.3)
2014	7847(27.9)
2015	7331(26.0)
2016	7759(27.5)

*UEBMI* Urban Employee Basic Medical Insurance scheme, *URBMI* Urban Resident Basic Medical Insurance scheme, *SD* Standard deviation

Table 3 shows the differences between hospital expenses and OOP expenses under the different payment methods. FFS had the highest hospital expenses (RMB12035.8/US$1889.6) and OOP expenses (RMB1734.3/US$272.3); per-diem payment had the lowest hospital expenses (RMB7496.0/US$1176.8); and CAP had the lowest OOP expenses (RMB1328.2/US$208.5). The reimbursement ratio, or OOP expenses as a proportion of total hospital expenses, of all the four payment methods exceeded 75%, with CAP having the highest reimbursement ratio (88.3%). CAP had the longest ALOS (15.4 days) and case-based payment (9.7 days) the lowest ALOS.

**Table 3:** Hospital and OOP expenses by payment methods

	Fee-for service	Per-diem payment	Capitation payment	Case-based payment	*P* value
Hospital expenses (RMB)					< 0.001
Median	12 035.8	7496.0	11 332.9	8583.9	
IQR	16 866.8	7189.5	15 012.8	14 028.0	
OOP expenses (RMB)					< 0.001
Median	1734.3	1506.7	1328.2	1412.0	
IQR	3118.8	1380.1	2061.2	2176.9	
Reimbursement ratio	85.6	79.9	88.3	83.6	
Average LOS	15.1	13.2	15.4	9.7	< 0.001

*IQR* Interquartile Range, *OOP* Out-of-pocket, *LOS* Length of hospital stay

*P* values are based on the Kruskal–Wallis test

**Fig. 1:**
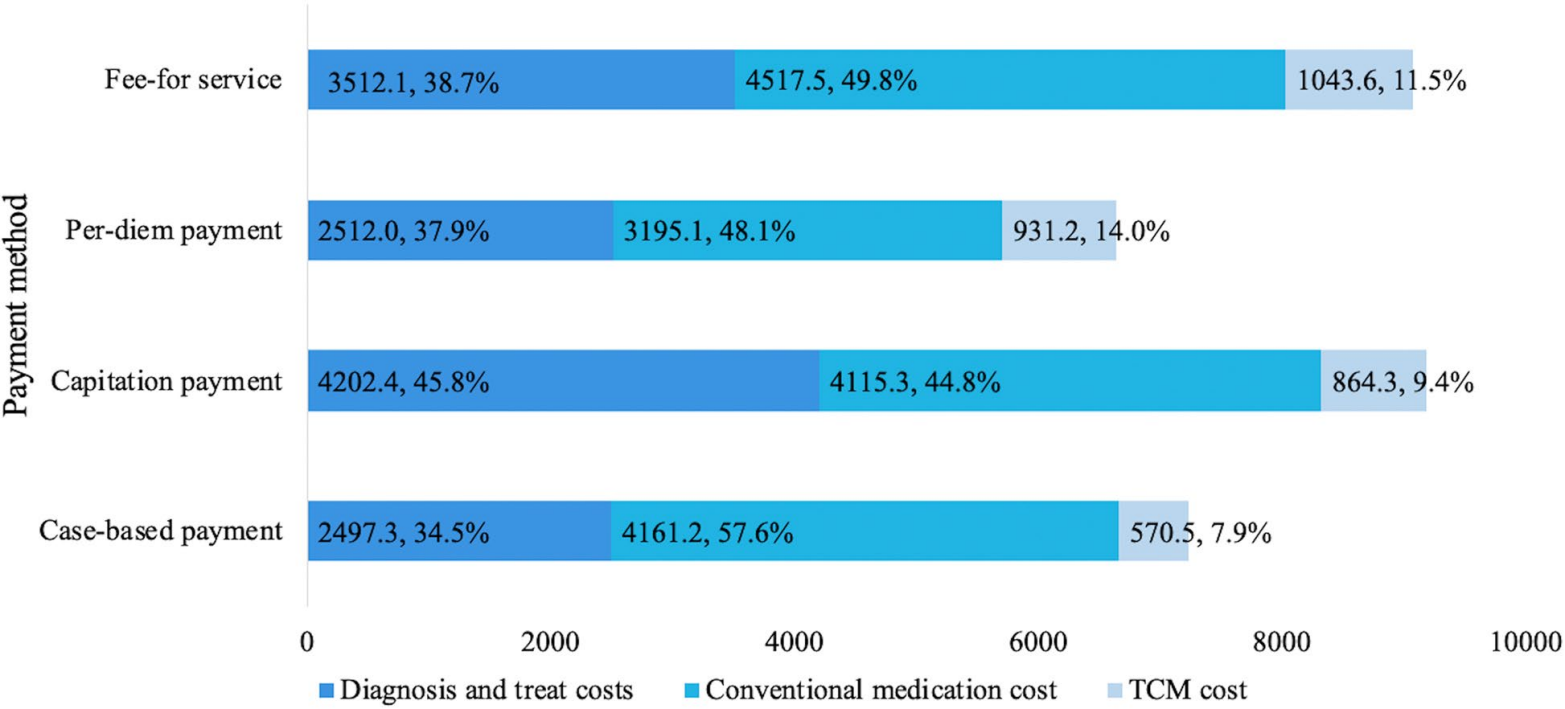
Composition of hospital expenses of lung cancer patients with different payment methods (RMB). TCM: traditional Chinese medicine; *P* < 0.001(based on the Kruskal–Wallis test)

Breaking down the three largest hospital expenses, Fig. 1 shows that CAP had the highest cost for diagnosis and treatment (RMB4202.4/US$659.8), followed by FFS (RMB3512.1/US$551.4) and per-diem payment (RMB2512.0/US$394.4). The conventional medication expenses (RMB4517.5/US$709.3) and TCM expenses (RMB1043.6/US$163.9) were highest in FFS; per-diem payment had the lowest expenses for conventional medicine (RMB3195.1/US$646.1); and case-based payment had the lowest expenses for TCM (RMB570.5/US$135.7).

Table 4 depicts the relationship between hospital expenses, OOP expenses and payment methods for lung cancer patients. After controlling for covariables, hospital expenses for per-diem payments were 21.3% lower (95% CI = -0.265, -0.215) and case-based payments were 8.4% lower (95% CI = -0.151, -0.024) than FFS. OOP expenses under per-diem payments were 9.2% lower (95% CI = -0.130, -0.063) and CAP was 15.1% lower (95% CI = -0.151, -0.024) than FFS, but case-based payment was higher than FFS.

**Table 4:** Association of inpatient and OOP costs with payment method through regression model

	Hospital expenses	OOP expenses
Ref: FFS		
Per-diem payment	-0.213^*^ (-0.265, -0.215)	-0.092^*^ (-0.130, -0.063)
Capitation payment	-0.013 (-0.044, 0.019)	-0.151^*^ (-0.204, -0.124)
Case-based payment	-0.084^*^ (-0.151, -0.024)	0.163^*^ (0.071, 0.231)

The control variables were FFS, and both models controlled for gender, medical insurance type, hospital level, number of comorbidities, region, and year

*OOP* Out-of-pocket, *FFS* Fee-for-service

^*^P < 0.05, the regression results (β) were converted by the formula: Coefficient = e^β^-1

## Discussion

We revealed significant differences in hospital and OOP expenses of lung cancer inpatients under China’s different medical insurance payment schemes. Specifically, the CAP was observed to generate the lowest OOP costs, but relatively high inpatient costs for patients compared to per-diem and case-based payments. The FFS was found to have both the highest hospital expenses and OOP costs. The per-diem payment method had the lowest hospital expense, with the OOP expenses ranking second among the four payment methods.

Our results show that the per-diem payment had not only the lowest cost of hospitalization, but also the lowest conventional medication expenses. In 2007, per-diem payment was implemented in China, with the aim of reducing the average cost of patients [25]. Per-diem payment in Japan reduced both inpatient medical charges and ALOS, but did not improve the quality of care [37]. The lowest OOP expenses for hospitalized patients was under CAP, which was consistent with previous findings that OOP expenses for patients can be reduced through CAP [38], without weakening the quality of medical care [24]. Yang et al. [15] argued that CAP won patient trust and reduced OOP expenses for patients. Table 3 shows that CAP had the second-highest hospital expenses, but the highest reimbursement rate under the four payment methods. Under CAP, a higher reimbursement rate meant a larger share of medical costs paid by the insurance scheme, where patients bore the least OOP expenses and health insurance funds faced a heavier burden of payment.

Per-diem and CAP had the greatest control over hospital expenses and largest reduction of OOP expenses for inpatients. This can be explained by the reimbursement standard of each unit under per-diem payments, which takes the severity of the patient's disease, ALOS, nursing level and surgical stage into account. Under per-diem and CAP, the actual medical expenses of patients with lung cancer could be accurately estimated by establishing reimbursement standards based on hospital historical data, specific to different regions and medical institutions [25]. The accurate estimation of medical costs contributed to reducing the burden of hospital expenses for lung cancer patients. Under CAP, hospitals were prepaid for a fixed fee and the estimated number of patients to be treated. Known as "special financial funds", about 50% of public health funding allocated by CAP are provided upfront, with further funds linked to measurable performance metrics, which reduces OOP expenses [37].

In addition, health care providers use their payment methods to incentivize particular hospital behavior, for example, a fixed value of reimbursement per unit of service under per-diem payments encourages hospitals to reduce costs per unit, lowering hospital expenses, but potentially leading to lower quality services [25]. Similarly, CAP without appropriate supervision and assessment could lead to low medical quality [39], when hospitals admit patients with minor medical conditions to reduce costs.

FFS was the most common payment method used by public hospitals, reimbursing hospitals for actual medical expenses incurred [40]. To obtain a higher reimbursement, hospitals might encourage over-servicing, where doctors provide more expensive drugs and diagnostic tests, resulting in higher medical expenses to insurers and patients [27, 35]. As shown in Fig. 1, medicine expenses were highest under FFS, where more than two-thirds of lung cancer patients were clinically diagnosed as advanced, resulting in intensive radiotherapy, chemotherapy, and other drugs treatments [41]. We speculate that under FFS, lung cancer patients could be over-treated. We were unable to measure the quality of care, but under FFS patients bore high OOP costs.

Our results also show that the hospital expenses and OOP expenses under pre-paid per-diem payment, and the hospital expenses under pre-paid case-based payment were lower than post-paid FFS. Jin et al. [30] reported that compared with FFS, case-based payment could reduce overtreatment and financial barriers to health care, and increase service quality as well. As a post-payment health insurance scheme, FFS placed significant financial risks on the patient [42]. We recommend one key approach to reducing patient OOP expenses is to establish pre-payment systems and move away from post-payment schemes in basic health insurance systems [43]. One constraint is that under pre-paid per-diem payment and case-based payment, hospitals tend to over-service, providing more treatments not covered by health insurance, the cost of which falls on patients as OOP expenses [44]. Under the pre-payment system, when hospitals generate excessive costs, the hospital bears part of the costs above NMIB reimbursements. The risk-adverse hospital will carefully control its costs, optimizing the medical service provision and minimizing unnecessary medical projects.

As shown in Table 4, OOP expenses under case-based payment were higher than FFS, which confirms findings by Jiang et al. [45, 46]. Another example is the China Center for Disease Control and Prevention (CDC) case-based payment to alleviate the financial burden of tuberculosis patients, which did not reduce patients’ OOP expenses as expected. In the CDC tuberculosis example, many medical services received by patients were not included in the standard treatment package, which increased OOP expenses. Table 3 showed that case-based payments had the shortest ALOS and the lowest hospital cost except for per-diem payments. To promote case-based payments, China has released a catalog of 320 diseases, including lung cancer [47], which encouraged clinical research and specified a series of standard treatment regimes. We recommend further testing of case-based payments for lung cancer sufferers.

When payment schemes minimize both hospital and OOP expenses, we consider it a good payment method [15]. However, none of the four payment methods satisfied this dual condition in our study. The payment methods under the post-payment system were more advantageous than pre-payment schemes in reducing hospital expenses. Pre-payment is subject to China’s current insurance payment reforms, which aim to reduce the use of FFS [48].

To continue poverty alleviation, China proposed a critical illness insurance system to reduce the OOP medical expenses burden of patients with critical illnesses [49, 50]. When the interests of patients and hospitals are in conflict, we recommend medical decision-makers select the insurance scheme based on the principle of maximizing the interests of patients. The CAP system maximized patient interests by minimizing OOP expenses for lung cancer patients. To balance the benefits of the medical insurance institutions, hospitals, and insured persons, our results for lung cancer patients recommend the case-based payment. Compared with CAP, while the OOP expenses of patients increased slightly under case-based payment, hospital expenses were greatly reduced, which was beneficial to insurers.

### Limitations

Our study has a number of limitations. First, our database does not include the discharge status of patients, so we cannot consider the quality of medical services or treatment outcomes. Second, since the database provides the main payment methods of lung cancer patients, it is impossible to rule out whether hospital used hybrid payment methods. Third, we did not study NCMS, covering rural residents. Future studies need to consider other cancers and other diseases to assesses whether the per-diem payment generated the lowest hospital expenses, while CAP had the lowest OOP expenses. Finally, data were not available after 2016, so DRG or URRBMI payment schemes were not studied. However, the UEBMI and URBMI schemes coverage and benefits have not changed significantly in the post-2016 period, and a major aim of URRBMI was to raise the rural NCMS benefits and coverage to URBMI levels.

## Conclusion

There was no single optimal payment method, with hospital and OOP expenses for lung cancer inpatients varying significantly between China’s payment regimes. We speculate that this also applies to other cancers and other diseases. Pre-paid schemes (per diem payments, case-based payments and CAP) were superior to post-paid FFS for lung cancer patients. Per-diem payment generated the lowest hospital expenses, while CAP had the lowest OOP expenses. We recommend that policymakers give priority to reducing patients' burden for major diseases and implement payment methods to reduce patients' OOP expenses. Case-based payments achieved a tripartite balance among minimizing expenses of medical insurers, controlling hospitals treatment costs, and protecting patient OOP expenses.

## Data Availability

Inpatient data were provided by the CHIRA, which is only available up to 2017 for researchers. The availability of these data is limited and can only be used by authorized institutions, so they cannot be disclosed. However, the researcher can make a reasonable application to CHIRA to obtain data. The data of this study cannot be disclosed due to authority factors.

## Abbreviations

CHIRA: China Medical Insurance Research Association
OOP: Out-of-pocket
FFS: Fee-for-service
CAP: Capitation payments
GLM: Generalized linear models
ALOS: Length of hospital stay
UEBMI: Urban Employee Basic Medical Insurance
URRBMI: Urban and Rural Residents Basic Medical Insurance
NMIB: National Medical Insurance Bureau
NCMS: New Rural Cooperative Medical System
DRG: Diagnostic-related group
LOS: Length of stay
TCM: Traditional Chinese medicine
SD: Standard deviation
IQR: Interquartile Range
CDC: China Center for Disease Control and Prevention
